# “Fake Tan” or “Fake News”?

**DOI:** 10.1177/2041669520915734

**Published:** 2020-04-11

**Authors:** Georg Meyer, Kinjiro Amano, Kaida Xiao, Sophie Wuerger

**Affiliations:** Department of Psychology, University of Liverpool; School of Medical Sciences, University of Manchester; School of Design, University of Leeds; Department of Psychology, University of Liverpool

**Keywords:** colour, perception, face perception, light, surfaces/materials

## Abstract

We estimated Trump’s skin colour from 70 internet images and also from the “twitter tan line” image (February 8, 2020; Twitter). We then compared the estimated skin colours with two existing data sets of skin colours: the range of skin tans that occur naturally in the Caucasian population and the range skin colours brought about by a sunless tan. We find that Trump’s skin colour is close to the edge of the natural skin tan gamut and firmly within the gamut of a sunless skin tan. The skin colour above Trump’s tan line is outside of the naturally occurring range of skin colours, even outside the skin tan of nonmelanized albinos. The latter finding is consistent with the hypothesis that part of the image may have been digitally distorted.

A striking picture of President Trump that shows a very distinctive “tan line” has been exciting the “twitter-sphere” (Figure 1). The purpose of this short report is to use existing data sets on skin colour to shed some light on the controversy surrounding this image. We are addressing both issues, fake news and fake tan.

**Figure 1.**
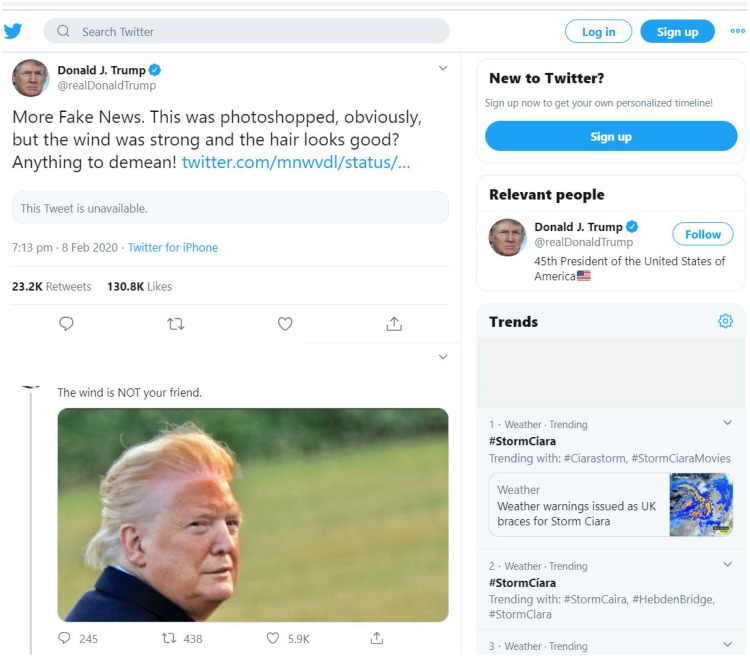
Controversial Image on Twitter Dated February 8, 2020.

Dihydroxyacetone (DHA), the primary ingredient used in most sunless tanning products, is known to lead to changes in skin colour (Wittgenstein & Berry, 1960). We measured this shift in skin colour in a Caucasian sample and then compared the resulting colours with the natural range of skin colours (Xiao et al., 2017). The two sets of data points are shown on the right side of Figure 2, together with the ellipses denoting a 95% confidence region, in a standard CIELAB colour space (Luo, 2016). Skin colour can be described by three values: L*, lightness—from black (0) to white (100); a*, green (−) to red (+); and b*, blue (−) to yellow (+). The space is designed to model our perceptual sensitivity to colour differences, taking into account the illumination. A good way to characterise skin tans is to look at L* and b* values, that is, skin lightness versus yellowness.

**Figure 2.**
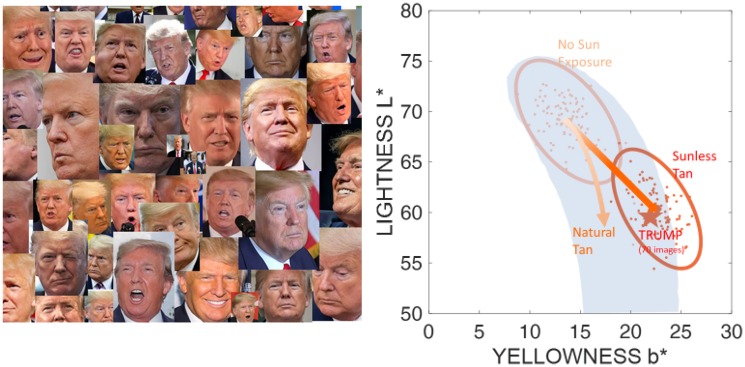
Internet Images Used to Estimate Trump’s Skin Colour. On the right side the skin colours for no-sun exposure and sunless tan are shown. The range of naturally occurring tans in the Caucasian population is indicated by the grey banana shape.

DHA very effectively changes skin colour; especially for users with initially quite light skin, the skin colour change measured at 24 hours after application follows a shift along a straight line towards dark and yellow. In Figure 2, the upper ellipse shows the original skin colour (“No Sun exposure”) and the lower right ellipse shows how the skin tone has changed 24 hours after application of DHA (“Sunless Tan”). The grey “banana” shape shows the naturally occurring skin tans in the Caucasian population, with the colour of a nonmelanized albino at the top (Chardon et al., 1991). Comparing the skin tones of our DHA sample with the banana shape shows that for a substantial part of our sample, the sunless tan is outside of the natural range. It is also noteworthy that the natural amount of yellowness depends on the lightness level and is maximal at a mid-lightness of 50 (b* = 25). For a given skin lightness, the average sunless tan is too yellow in comparison to the natural tan. The arrows in Figure 2 (right side) indicate the direction of colour change brought about by natural tanning (on the left) or by sunless tanning (on the right).

## Question 1: Fake Tan?

There is no shortage of recent images of President Trump on the internet. These images, however, will have been taken in many different environments with different ambient illuminations and may well have been significantly altered by the authors. To estimate Trump’s real skin colour, we downloaded 70 recent images from the internet and sampled five randomly selected points in the region around his forehead in each image (Figure 2, left side). Trump wears a white shirt in most images, which enables us to roughly compensate for differences in illumination by estimating the “white point” for each image. As for the forehead, five samples of the white shirt were randomly selected. From these RGB data (all images were 8-bit jpeg images and we assumed sRGB), L*a*b* values were computed and plotted on our graph of skin tans (Figure 2, right side). Trump’s forehead (star labelled “TRUMP”; L*a*b* coordinates: 59.57, 21.12, 21.94) lies well within the skin colours that we expect to see 24 hours after the application of an 8% DHA sunless tanning product. Is the tan fake? Well, we will never know, but a fake tan would look like Trump’s forehead.

## Question 2: Fake News?

**Figure 3.**
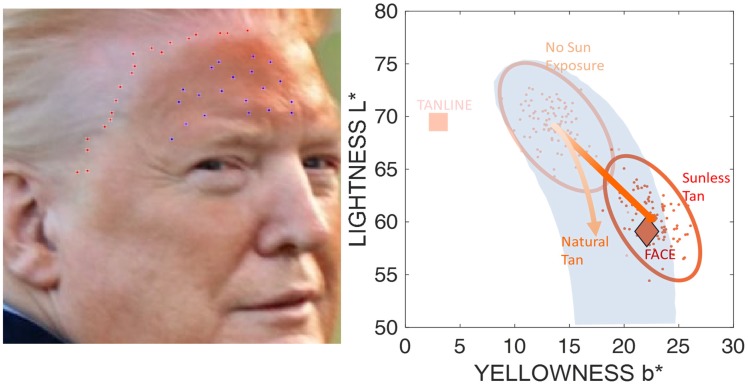
The Image on the Left Is Used to Estimate the Colour Above (Red Dots) and Below the Tan Line (Blue Dots). The figure on the right shows the estimated skin colour in the L*b* diagram.

The “tan line” picture (Figure 3, left) is one of the relatively few images of the President where no white shirt is visible, so we have to assume a “white point.” Since the picture was taken outside, the “D65” (standard “daylight”) and “D50” (horizon light—early morning or late evening light) illuminants were assumed. Since the resulting estimates were very similar, we only report D50.

We randomly sampled the “tan line” images to obtain 20 datapoints in the “tanned” part of the forehead and temples (see blue dots in Figure 3) and another 20 datapoints above the “tan line” portion of the image (red dots). The average values for both sets of measurements were, again, plotted against our standard data.

The average L*b* coordinates below the “tan line” (labelled “FACE” in Figure 3, right side) were very similar to the average of the estimates over all internet images of the president (Lab [D50]: 59.10, 25.40, 22.07). The skin colour in the image is therefore very similar to the colour shown in most other images. The average data for the samples taken from “above the tan line” (labelled “TANLINE”; L*a*b* [D50] = 69.48, 11.77, 3.01) are well outside the “natural range” seen in Caucasians, even in nonmelanized albinos. Is the picture fake? We may never know—the estimated face colour in the image is quite similar to that seen in other images (see Figure 2). The colour of the light skin above the “tan line,” however, is strikingly unnatural.

In conclusion, we estimated Trump’s skin colour from 70 internet images and the “twitter tan line” image. The agreement between these two sets of estimates is quite remarkable which gives us some confidence that our estimates are reasonably reliable. Subject to our assumptions made about the conversion from RGB to device-independent colours, we draw the following cautious conclusions: (a) Our analysis puts Trump’s skin tan close to the edge of the natural skin tan gamut and firmly within the gamut of a sunless skin tan. (b) The estimated skin colour above Trump’s tan line is outside of the naturally occurring range of skin colours, even outside of the range of skin tans of nonmelanized albinos, consistent with some image colour distortions.
